# Optimization and Corroboration of the Regulatory Pathway of p42.3 Protein in the Pathogenesis of Gastric Carcinoma

**DOI:** 10.1155/2015/683679

**Published:** 2015-05-28

**Authors:** Yibin Hao, Tianli Fan, Kejun Nan

**Affiliations:** ^1^Zhengzhou Central Hospital, Zhengzhou, Henan 450007, China; ^2^Department of Pharmacology, School of Basic Medicine, Zhengzhou University, Zhengzhou, Henan 450001, China; ^3^Department of Oncology, The First Affiliated Hospital, College of Medicine of Xi'an Jiaotong University, Xi'an, Shaanxi 710061, China

## Abstract

*Aims*. To optimize and verify the regulatory pathway of p42.3 in the pathogenesis of gastric carcinoma (GC) by intelligent algorithm. *Methods*. Bioinformatics methods were used to analyze the features of structural domain in p42.3 protein. Proteins with the same domains and similar functions to p42.3 were screened out for reference. The possible regulatory pathway of p42.3 was established by integrating the acting pathways of these proteins. Then, the similarity between the reference proteins and p42.3 protein was figured out by multiparameter weighted summation method. The calculation result was taken as the prior probability of the initial node in Bayesian network. Besides, the probability of occurrence in different pathways was calculated by conditional probability formula, and the one with the maximum probability was regarded as the most possible pathway of p42.3. Finally, molecular biological experiments were conducted to prove it. *Results*. In Bayesian network of p42.3, probability of the acting pathway “S100A11→RAGE→P38→MAPK→Microtubule-associated protein→Spindle protein→Centromere protein→Cell proliferation” was the biggest, and it was also validated by biological experiments. *Conclusions*. The possibly important role of p42.3 in the occurrence of gastric carcinoma was verified by theoretical analysis and preliminary test, helping in studying the relationship between p42.3 and gastric carcinoma.

## 1. Introduction

The occurrence and development of gastric carcinoma is a multifactor, multistage, and multistep process [1]. A large number of molecules have been involved in it and constituted a complex regulatory network [2]. Finding and identifying the key biomarkers of high-risk warning, early diagnosis, and effective treatment of gastric cancer are a focus of gastric cancer research [3]. So far, studies have confirmed that multiple antioncogenes such as PTEN [4], p16 [5], p21 [3], Smad4 [2], Fas [6], and RECK [3] and oncogenes such as, Ras [7], c-myc [1], and MMPs [8] are associated with the development of gastric carcinoma. p42.3 is a novel gene, cloned by applying synchronization, mRNA differential display and bioinformatics. Researchers have proved that p42.3 may play a vital role in the occurrence and development of gastric carcinoma [9]. Some studies indicate that p42.3 has the characteristic of oncogenes and tumor markers and it may be one of the early molecular events in the development from gastric mucosa lesion to gastric carcinoma [10]. However, these results did not explain systematically the specific function of p42.3 in it.

According to our early study, p42.3 may be involved in the regulatory pathway in the occurrence and development of gastric carcinoma and the regulatory pathway is as follows: Ras→Raf-1→MEK→MAPK kinase→MAPK→microtubule-associated protein→spindle protein→centromere protein→cell proliferation [11]. Nevertheless, it has not been verified by molecular biological experiments. On the basis of our previous study, through improvement of the similarity algorithm between the reference protein and p42.3 protein, this study investigated the biological features of p42.3 by means of the regulatory network of the reference protein, optimized the regulatory network of p42.3, modulated the maximum possible pathway correspondingly, and verified it by preliminary molecular biological experiments.

## 2. Materials and Methods

### 2.1. Materials

Gastric carcinoma cell lines BGC823, MGC803, SGC7901, AGS, N87, and GES1 were provided by Beijing Cancer Hospital (the original sources were from Shanghai Bioleaf Biotech Co., Ltd.) and were cultivated in DMEM culture media with 5% fetal bovine serum in a 5% CO_2_ cell culture box at 37°C.

### 2.2. Methods

#### 2.2.1. Structural Features of p42.3

After obtaining the amino acid sequence of p42.3 (GenBank: NP_848543) from NCBI database, the spatial structure of protein was predicted by the threading prediction tool Phyre^2^ (http://www.imperial.ac.uk/phyre/, Imperial College London) [12]. Then, relevance to cell proliferation in terms of function was set as the restrictive condition, based on which the protein with the two structural domains were searched and constituted the data set of reference proteins. Whereby, the possible biological property of p42.3 was studied.

#### 2.2.2. Similarity Calculation of the Reference Protein and p42.3 Protein

Multiparameter weighted sum method was put to use in calculating the similarity of reference protein and p42.3. First select several parameters in which the two proteins have similarity to calculate the degree of similarity of each parameter, and then add the weight trained by artificial neural network. Finally, obtain the degree of similarity after a weighted summation.

#### 2.2.3. Selection of the Parameters

According to the literature data, the following nine parameters of protein similarity were selected: protein spatial structure, the number of atoms inside the molecule, the number of amino acids in each protein, the species of amino acids, the location of element P and element S in the protein molecule, and the proportion of the number of atoms C, N, and O in the protein molecule [13–16].

#### 2.2.4. Similarity Calculation of the Spatial Structure of Protein

Before calculating similarity values, the coordinates of each atom in the protein structure file (pdb file) were determined and Euclidean coordinates were used as spatial coordinates, with the geometrical center of the protein as the origin. The distance from each atom to the origin was then calculated. According to these distances, the protein was divided into layers and the structure similarity of two proteins in corresponding layers was analyzed by stratified analysis. It was found that the distances between most of the atoms of p42.3 protein and the origin were in the range of 0~80 nm and a small portion of the distance were within 80~100 nm, and also, very few of them were above 100 nm. Therefore, based on the length of radius, p42.3 protein was divided into 10 layers from the center to outer edge. The distances of each layer were as follows: the first layer 0~10 nm; the second layer 10~20 nm; the third layer 20~30 nm; the fourth layer 30~40 nm; the fifth layer 40~50 nm; the sixth layer 50~60 nm; the seventh layer 60~70 nm; the eighth layer 70~80 nm; the ninth layer 80~100 nm; and the tenth layer beyond 100 nm. The number of atoms in each layer was counted for each of the proteins being compared and stored in array vector data 1 and data 2, respectively. The similarity in atom numbers in each layer was then compared using the formula: sim = 1 − (|data 1 − data 2|/data 1), wherein sim represents a ten-dimensional vector that has stored the similarity of each layer.

Weights were then added to the similarity of each layer and the overall density similarity was calculated by the weighted summation method. It is reasonable to suppose that the layers that contain the most atoms will be more likely to determine properties of the protein. Based on this assumption, the more atoms the layer owns, the higher the weight of this layer is, so the proportion of the atoms number in each layer determined the weight of this layer. Of course, it is maybe different in every layer for two proteins, so the average would be taken. Hence, each layer was weighted as the following formula: *w*_*i*_ = ((*l*_1*i*_/*n*_1_) + (*l*_2*i*_/*n*_2_))/2, *i* = 1,2,…, 10, where *n*_1_ is the total number of atoms of the first protein, *n*_2_ is the total number of atoms of the second protein, while *l*_1*i*_ and *l*_2*i*_ are the number of atoms in the *i*th layer in protein 1 and protein 2, respectively. Thus, the spatial structure similarity of the two proteins was obtained.

#### 2.2.5. Similarity of the Total Number of Atoms and the Number and Type of Amino Acids

Similarity algorithms of the three parameters were alike. The number of atoms and amino acids, and the number of amino acid types in the two proteins were calculated by textread function in MATLAB software. Then, the number of atoms and the number and type of amino acid can be read from the pdb file of the two proteins. The total number of atoms of the two proteins was recorded as *n*_1_ and *n*_2_, respectively, and then the formula used to calculate the similarity in atom numbers was sim_*a*_ = 1 − (|*n*_1_ − *n*_2_|/*n*_1_). Likewise, the similarity of the number of amino acids and its types could be also obtained.

#### 2.2.6. Similarity of Each Element

This study was mainly to analyze elements C, N, O, P, and S. Firstly, the proportion of the number of C, N, and O to the total number of atoms in each protein was calculated. Then, the similarity was calculated among C, N, and O in accordance with the formula: sim_element = 1 − (|*n*_1_ − *n*_2_|/*n*_1_). In addition, in protein molecules, the number of elements P and S was usually small, but they both play crucial roles in the function of protein. While in p42.3 protein, there was only one S atom and no P atoms. Therefore, it is obviously not scientific to calculate the degree of similarity according to the number of atoms of the two elements. Instead, similarity of the location between atoms P and S was set as the criteria for calculation. In this algorithm, it was assumed that if the two elements P and S were in the same layer, the similarity was regarded as 1.0; if they were in adjacent layers, the similarity was 0.8; otherwise, the similarity was 0. Therefore, the similarity parameter of each element in proteins was achieved.

#### 2.2.7. Calculation of the Weight of Each Parameter

Based on the similarity of each parameter of protein that had been figured out, the overall similarity was worked out by the weighted summation method. Before this, data of 100 pairs of similar protein pairs had been collected. According to the methods described above, the similarity of each parameter in each pair of proteins had been calculated: *S*1–*S*9. Then, BLASTp was used to search the homology of each pair of proteins, which was regarded as the overall similarity. Therefore, for each pair of proteins, a similarity data vector of 1*∗*10 can be achieved: [*S*1, *S*2, *S*3, *S*4, *S*5, *S*6, *S*7, *S*8, *S*9, *S*]. Then the similarity data of the 100 pairs of proteins was input to BP (back propagation) artificial neural network for training; thus, the weights of each parameter Qi had been achieved (Table 1).

Therefore, for each pair of proteins, their overall similarity can be calculated by formula: *S* = 0.3183*S*_1_ + 0.0343*S*_2_ + 0.0204*S*_3_ + 0.0603*S*_4_ + 0.0653*S*_5_ + 0.1062*S*_6_ + 0.1002*S*_7_ + 0.1477*S*_8_ + 0.1480*S*_9_. In this formula, *S* is the overall similarity of the two proteins. *S*_*i*_ represents the similarity of each parameter. *i* = 1,2 …, 9 were spatial structure (density), number of atoms in the protein, number and type of amino acids, number and proportion of C, N, and O atoms [8], and spatial position of P and S atoms in the protein, respectively. On the basis of this formula, the similarity of the reference protein and p42.3 was thus figured out and the data set of the reference proteins was composed.

#### 2.2.8. Construction and Optimization of a Bayesian Regulatory Network

In condition of cellular proliferation, the reference protein set obtained by the similarity calculation was screened out. Then, with a reference protein as the starting point and cell proliferation as the ending point, the acting pathway and node of each reference protein were collected. There are crosses between different pathways, thus constituting a regulatory network [11]. In the network, “+” indicates a positive role in promoting the regulation; “−” represents a negative role in inhibiting the regulation. The similarity of each reference protein and p42.3 was set as the initially prior probability. By applying knowledge of conditional probability, the probability of occurrence in each node was worked out. The formula is

(1) $$\begin{array}{c} P\left(E\right)=P\left(\bar{A}\;\bar{B}C\right)+P\left(\bar{A}\;\bar{B}D\right)+P\left(\bar{A}\;\bar{B}CD\right). \end{array}$$

Bayesian networks are Directed Acyclic Graphs (DAGs), which describe the joint probability distribution of a finite set of variables *U* = {*X*_1_, *X*_2_,…, *X*_*n*_}. Bayesian networks can be symbolized by the element pair *B* = (*G*, *θ*), where *G* is a DAG in which the nodes represent random variables *X*_1_, *X*_2_,…, *X*_*n*_. It can symbolize gene expression vectors in expression profiling data, while *θ* represents the conditional probability of each variable. DAG showed the independent relation under the following conditions. It was the Markov assumption; each variable *X*_*i*_ was independent of its nonchild node in the prerequisite that it was the parent node in *G*. Based on the assumption of independence, the Bayesian network *G* had only one joint probability distribution for set *U* was *P*(*X*_*i*_,…, *X*_*n*_) = ∏_*i*=1_^*n*^ *P*  (*X*_*i*_∣Pa(*X*_*i*_)), where Pa(*X*_*i*_) symbolizes the parent node of *X*_*i*_. In order to determine the joint probability above, all the conditional probabilities in this formula need to be confirmed.

In the Bayesian network in this paper, after obtaining the probability of occurrence in each node and pathway, the Bayes theorem was used to inverse the probability of protein in acting their roles in each node, thus finding the highest possible regulatory pathway of p42.3 protein.

#### 2.2.9. The Molecular Biological Test of the Optimal Path

After obtaining the highest possible acting pathway of p42.3 protein through calculation and prediction, some basic biological experiments were carried out for initial validation. To begin with, Trizol (invitrogen, America) method was used for extraction of the total mRNA in the six cell lines: BGC823, MGC803, SGC7901, AGS, N87, and GES1. Through reverse transcription, cDNA was synthesized (cDNA reverse transcription kit, Thermo fisher scientific company, United Kingdom). According to the gene sequence of the reference proteins and p42.3, the primers were designed. The sequence of the primers was shown in Table 2. In contrast with *β*-actin, the RT-PCR was used to amplify, respectively, (PCR amplifier, Eppendorf Company, Germany). After PCR products were detected by agarose gel electrophoresis, the expressions of various proteins in different cell lines were compared.

## 3. Results

### 3.1. Structural Features of the EF-Hand and CC-Domain

**Figure 1:**
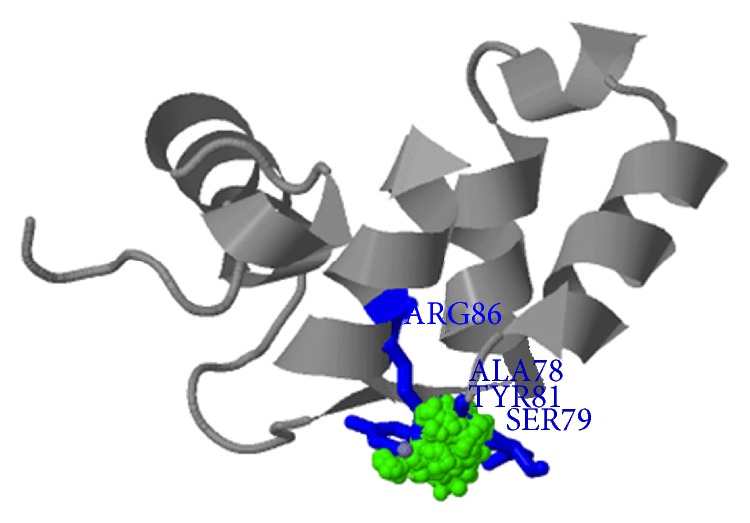
Ligand combination model of EF-hand structural domain in p42.3 molecule.

The spatial structure of protein was predicted by the threading prediction tool Phyre. A three-dimensional ligand-binding model of the characteristic of p42.3 in EF-hand region was predicted by using 3DLigandSite (http://www.sbg.bio.ic.ac.uk/~3dligandsite/, Imperial College London) [17]. The metal ion binding sites of p42.3 were ALA78, SER79, TYR81, and ARG86, as shown in Figure 1. The protein data set that had high structural homology with EF-hand and CC-domain (p42.3 molecule) was searched. Some of them with the same structure of EF-hand were shown in Table 3. Then, proteins relating to cell proliferation functionally were screened out as the reference protein.

### 3.2. Similarity Calculation of the Reference Protein and p42.3

The similarity algorithm of protein was compared by the similarity of nine parameters mentioned above. The MATLAB software (MathWorks, America) was used for programming. The similarity of the reference protein and p42.3 was calculated. After screening by “cell proliferation,” the results were displayed in Table 4.

### 3.3. Bayesian Regulatory Network

**Figure 2:**
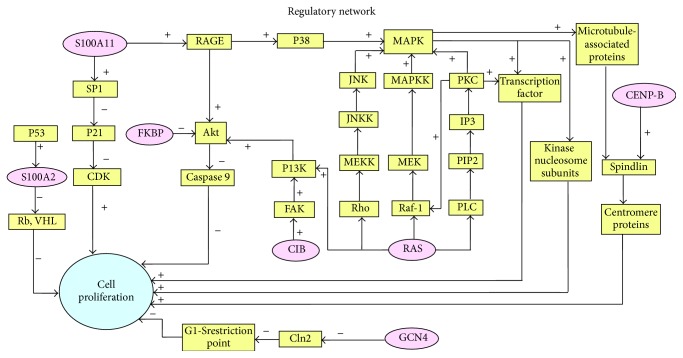
Primary regulatory networks.

Cell proliferation was set as the restrictive condition, and the acting pathways and nodes of different reference proteins were worked out by literature collection. With different acting pathways crossing, the regulatory network was thus formed, shown in Figure 2. The round nodes represent the reference proteins and they are the initial nodes. The relation between each node is upstream and downstream regulation. Arrows indicate the direction of action. “+”: a positive regulation and “−”: a reverse regulation.

**Figure 3:**
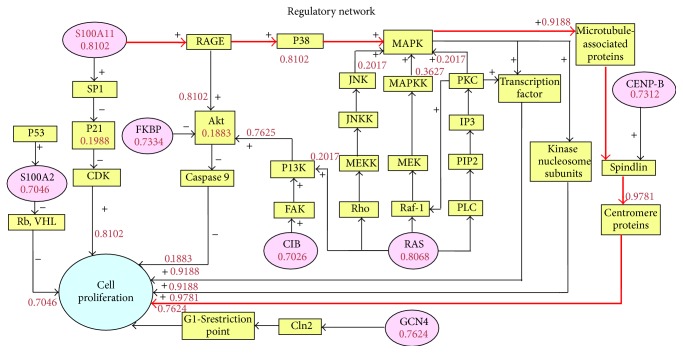
The most possible acting pathway of p42.3 protein by optimization of Bayes theorem.

The similarity of the reference proteins and p42.3 was treated as prior probability of the initial parent nodes. According to formula (1), the probability of occurrence in each node downstream was calculated until figuring out the final results of cell proliferation. The final one is the probability of occurrence of that pathway. The results were shown in Figure 3. The probability of the path in thick line was 0.9781, higher than that of other pathways. Connected with the results of protein similarity comparison, it can be initially verified that the pathway “S100A11→RAGE→P38→MAPK→Microtubule-associated protein→Spindle protein→Centromere protein→Cell proliferation” was with the highest possibility.

### 3.4. The Molecular Biology Test

**Figure 4:**
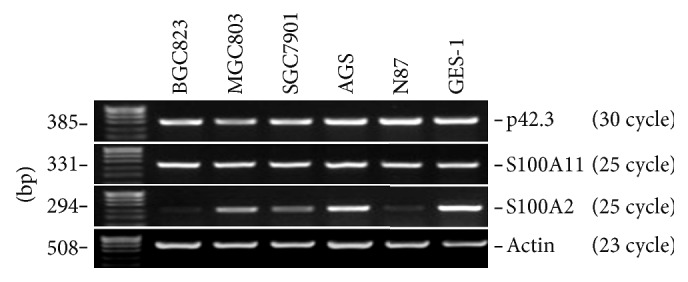
Expressions of p42.3, S100A11, and S100A2 in the cell lines of gastric carcinoma.

Based on the analysis of the spatial structure of p42.3 and Bayesian regulatory network, expressions of S100A11 (the protein with the largest positive maximum weighted value) and S100A2 (the protein with the shortest negative acting path) in gastric carcinoma cell lines were examined, respectively, for preliminary valediction of the correlation of p42.3 and S100A11. The results showed that when p42.3 showed normal expression, both S100A11 and S100A2 had shown expressions. In Figure 4, it was indicated that expression of S100A11 was extremely similar to that of p42.3, while the expression of S100A2 was considerably different from that of p42.3. By referring to the analysis of the protein structure, it could be concluded that the regulatory pathway of p42.3 may be consistent with that of S100A11, or it may be involved in the regulatory pathway.

## 4. Discussion

The occurrence and development of gastric carcinoma involve changes in the structure and expression of multiple related genes [9]. In particular, the activation of oncogenes and inactivation of tumor suppressors play important roles in it [10]. So far, many studies have tried to disclose the molecular regulatory mechanisms of gastric carcinoma in order to find biomarkers for the diagnosis and treatment of gastric cancer, which is expected to be an effective adjuvant therapy of surgery and chemoradiotherapy.

p42.3 expression is dependent on mitosis and is expressed at low levels or not at all in normal gastric mucosa but is highly expressed in gastric carcinoma tissues. It has the effect of promoting cellular proliferation and tumor metastasis [9]. Changes of p42.3 gene expression that occur during the development of gastric carcinoma indicate that p42.3 might be a direction of gastric carcinoma diagnosis and treatment [10, 11]. It was found that an EF-hand structural domain existed in the N-terminal amino acid sequence of p42.3 protein, which also presented in the S100 family of proteins [36]. The EF-hand structure consists of a typical helix-loop-helix structural unit; that is, two alpha helixes linked by a Ca^2+^ chelate ring [37]. In all the reports about EF-hand structures, the majority of EF-hand structural domains are even number and form structural domain pairs, separated by connexin, or homologous or heterologous dimmers, such as S100 family proteins with two EF-hand structural domains [38, 39]. Proteins with odd number of structural domains usually need to form homologous or heterologous dimers and their activity is presented in the form of dimer. CC-domain is a kind of super-secondary structure of protein, intertwined by two to seven *α* helices (most commonly two or four) to form a braided structure [40]. Many proteins with coiled helical structures have significant biological functions, such as the transcription factor in the regulation of gene expression [40]. The most well-known proteins containing coiled helical structures are oncoprotein and tropomyosin. To study the action mechanism of p42.3, a similarity algorithm with multiparameter calculation was adopted to find proteins with high structural similarity to p42.3. As a result, proteins that might be related to the occurrence of gastric carcinoma were screened and treated as gene regulatory path nodes [11]. By a series of probability calculation, it was found that the possible action mechanism of p42.3 in the pathogenesis of gastric carcinoma was S100A11→RAGE→P38→MAPK→Microtubule-associated protein→Spindle protein→ Centromere protein→Cell proliferation (Figure 3). And the initial molecule experiments also confirmed the consistency of p42.3 and S100A11 gene expression in gastric carcinoma cell (Figure 4). The study of gene regulatory networks can be used to quantitatively mine information regarding gene expression regulation from one side. Through extracting and analyzing this information, gene function and genetic networks can be understood, and the pathogenesis of the disease will be clear. The study of gene regulatory networks aids in the exploration of gene function in the overall framework [11]. Genes' functions should be studied not only from a structural level but also from a network level. Genes affect each other and work together in intricate networks, which consequently contain new functions that cannot be fully revealed by the DNA sequence.

The S100 proteins are a group of calcium-binding proteins with low molecular weight (10–12 kDa). Its amino acid sequence is highly conserved in vertebrates [41]. S100 proteins share a high degree of homology with calmodulin and other EF-Hand calcium binding proteins [41]. From the biological function, specific expression and chromosomal localization in tumor of S100 protein family and the intimate relation between S100 protein and tumor can be found. Recently, studies have indicated that S100A11 (S100C) can serve as a tumor suppressor protein in some tumors and a tumor promoter in other tumors [42]. S100A11 is upregulated in breast cancer, prostate cancer, and nonsmall cell lung cancer, where it promotes tumor metastasis and invasion [43, 44]. On the contrary, S100A11 acts as a tumor suppressor in urinary bladder and renal carcinoma [45]. Our experimental results present an upregulated expression of S100A11 in gastric cancer. As a candidate tumor suppressor protein, the expression of S100A2 is significantly lower in a variety of malignancies, such as breast, liver, prostatic, and esophageal cancer [46–49]. Studies have indicated that S100A2 can inhibit cell proliferation and invasion and act as a tumor suppressor involved in the occurrence and metastasis of gastric carcinoma [50], which is in agreement with our findings. Through analysis of expression of S100A11 and S100A2 in gastric cancer, both of which contained EF-Hand structure, it was verified that p42.3 could participate in the occurrence and development of gastric cancer from both consistent and opposite to the p42.3 effect direction.

Currently, there are various ways to compare protein structures, each with their own advantages and disadvantages [51]. By analyzing the structure of the proteins, most of them calculate the similarity value of a pair of proteins by applying a mathematical algorithm. That is, from the spatial conformation of protein, they all have only analyzed the characteristics of spatial structure of proteins. The similarity of proteins in other aspects was not taken into account. For example, element P and element S are crucial to the functions of proteins. Using the multiparameter comprehensive comparison method, this study not only compared the differences between the two proteins in the spatial atomic density but also considered the similarity of many other aspects. When conducting the weighted summation of each parameter, the weights used all came from training of diverse data not from subjective weighting. It guarantees the accuracy of weight of each parameter and avoids the mistakes that some parameter is of little importance to the overall similarity but with high weight. Consequently, the similarity of two proteins was figured out more accurately. All the process of calculation was carried out by the M file compiled by MATLAB. Batch comparison of any amount of proteins could be carried out easily and quickly.

## 5. Conclusions

Here, the ligand-binding model of the EF-hand structure of p42.3 was successfully predicted. Meanwhile, a Bayesian network using the corresponding mathematical algorithm was constructed and optimized to predict the most likely pathway. On the other hand, molecular biology experiments indicated that p42.3 and S100A11 may be with the commonplace in character, and this provided a hypothesis for us to conduct further research. In a word, our findings provide important research directions for exploring the mechanism of action of p42.3 in gastric cancer.

## Figures and Tables

**Table 1:** Similarity weights for each parameter.

Parameter	Similarity weight
Q1: Protein density	0.3183
Q2: Total number of atoms in each proteins	0.0343
Q3: Number of amino acids	0.0204
Q4: Amino acid type	0.0603
Q5: C	0.0653
Q6: N	0.1062
Q7: O	0.1002
Q8: P	0.1477
Q9: S	0.1480

**Table 2:** Primers sequence of PCR.

Name	Primer sequence	Amplification length	Renaturation temperature
S100A11	F: 5′-ATCGAGTCCCTGATTGCTGT-3′	331 bp	59°C
	R: 5′-AGAAAGGCTGGAAGGAAAGG-3′		

S100A2	F: 5′-CGCGAATTCATGTGCAGTTCTCTGGA-3′	294 bp	56°C
	R: 5′-CCGGGATCCCTCAGGGTCGGTCTGG-3′		

*β*-actin	F: 5′-TTCTGACCCATACCCACCAT-3′	508 bp	56°C
	R: 5′-ATTACAGTGCGTGCTAAAGG-3′		

**Table 3:** Structural data set of EF-hand that is similar to the partial structure of p42.3 molecule.

SCOP code	*E* value	Estimated precision	Fold/PDB descriptor	Superfamily	Family
d1iq3a [18, 19]	0.51	80%	EF hand-like	EF-hand	EH domain
d1s6ja [20, 21]	0.52	80%	EF hand-like	EF-hand	Calmodulin-like
c2pmyB [22, 23]	0.61	80%	PDB header: structural genomics, unknown function	PDB molecule: ras and EF-hand domain-containing protein	PDB title: EF-hand domain of human rasef
d1sw8a [24–26]	0.71	75%	EF hand-like	EF-hand	Calmodulin-like
d1c7va [27, 28]	0.76	75%	EF hand-like	EF-hand	Calmodulin-like
d1hqva [29]	0.85	75%	EF hand-like	EF-hand	Penta-EF-hand proteins
d1tiza [30, 31]	0.89	75%	EF hand-like	EF-hand	Calmodulin-like
d1g33a [32, 33]	0.91	75%	EF hand-like	EF-hand	Parvalbumin
d1fw4a [34]	1	75%	EF hand-like	EF-hand	Calmodulin-like
d1f54a [35]	1	75%	EF hand-like	EF-hand	Calmodulin-like

**Table 4:** Protein data set gained by using the spherical coordinate space hierarchical similarity algorithm.

Protein name	Number of atoms	Number of amino acids	Type of amino acid	C	N	O	S	P	Spatial structure	Overall similarity
Weight allocation	0.0343	0.020	0.0603	0.0653	0.1062	0.1002	0.1480	0.1477	0.3183	—
S100A11	0.9708	0.9083	0.5294	0.9704	0.8677	0.9226	0.8000	1.0000	0.7384	0.8102
RASEF	0.6557	0.6881	0.9412	0.9929	0.9497	0.9069	0.8000	1.0000	0.6515	0.8068
GCN4	0.6241	0.2752	0.8235	0.9615	0.9271	0.9972	0.8000	1.0000	0.6231	0.7624
FKBP	−0.0255	0.9817	0.7647	0.9655	0.9067	0.9589	1.0000	1.0000	0.5055	0.7334
CENP-B	0.1144	0.4587	0.7647	0.9478	0.9172	0.9488	1.0000	1.0000	0.5535	0.7312
S100A2	0.2032	0.8532	0.5882	0.9611	0.8015	0.8642	0.8000	1.0000	0.5756	0.7046
CIB	0.1034	0.0826	0.8235	0.9497	0.8854	0.9778	0.8000	1.0000	0.5765	0.7026
GPD1	−0.5864	0.6789	0.7647	0.9561	0.8754	0.9505	0.8000	1.0000	0.5905	0.6944
PAK1	0.1521	−0.0275	0.6471	0.9575	0.8543	0.9398	0.8000	1.0000	0.5647	0.6716
ACTN1	−0.2701	−0.3761	0.8235	0.9596	0.9004	0.9721	1.0000	1.0000	0.5902	0.6883
APC	0.9720	0.5046	1.0000	0.9902	0.8994	0.8022	0	1.0000	0.4929	0.6709
GP41	0.4489	0.4128	0.7647	0.9504	0.9527	0.9699	0	1.0000	0.4517	0.6166
S100A12	0.2007	0.8257	0.2941	0.9603	0.8809	0.9300	0	1.0000	0.5668	0.6058
MACF	−0.7944	−0.3578	0.5294	0.9641	0.8395	0.8972	0.8000	1.0000	0.4992	0.5691
MST3	−0.4866	−0.5963	0.2941	0.9407	0.8296	0.9467	0	1.0000	0.5876	0.4997
CHP1	−1.7798	0.1376	0.8824	0.9796	0.9118	0.9021	0	1.0000	0.4568	0.4969
S100A1	−1.5122	0.8532	0.8824	0.5230	0.4119	0.5927	1.0000	1.0000	0.3412	0.4923
